# When mechanical engineering inspired from physiology improves postural-related somatosensory processes

**DOI:** 10.1038/s41598-023-45381-z

**Published:** 2023-11-09

**Authors:** Chloé Sutter, Marie Fabre, Francesco Massi, Jean Blouin, Laurence Mouchnino

**Affiliations:** 1 Laboratoire de Neurosciences Cognitives, FR 3C, Aix-Marseille Université, CNRS, 3 Place Victor Hugo, 13331 Marseille, France; 2 Dipartimento di Ingegneria Meccanica ed Aerospaziale, Università degli Studi di Roma «La Sapienza», Rome, Italy; 3 Laboratoire de Mécanique des Contacts et des Structures, Institut National des Sciences Appliquées de Lyon (INSA LYON), Lyon, France; 4 Institut Universitaire de France, Paris, France

**Keywords:** Sensorimotor processing, Sensory processing, Touch receptors

## Abstract

Despite numerous studies uncovering the neural signature of tactile processing, tactile afferent inputs relating to the contact surface has not been studied so far. Foot tactile receptors being the first stimulated by the relative movement of the foot skin and the underneath moving support play an important role in the sensorimotor transformation giving rise to a postural reaction. A biomimetic surface, i.e., complying with the skin dermatoglyphs and tactile receptors characteristics should facilitate the cortical processes. Participants (n = 15) stood either on a biomimetic surface or on two control surfaces, when a sudden acceleration of the supporting surface was triggered (*experiment* 1)*.* A larger intensity and shorter somatosensory response (i.e., SEP) was evoked by the biomimetic surface motion. This result and the associated decrease of theta activity (5–7 Hz) over the posterior parietal cortex suggest that increasing the amount of sensory input processing could make the balance task less challenging when standing on a biomimetic surface. This key point was confirmed by a *second experiment* (n = 21) where a cognitive task was added, hence decreasing the attentional resources devoted to the balance motor task. Greater efficiency of the postural reaction was observed while standing on the biomimetic than on the control surfaces.

## Introduction

During everyday life, unpredictable circumstances can challenge our equilibrium while standing. This occurs for example when standing passengers are subjected to unexpected acceleration (e.g., braking manoeuvres in public transport). Exquisitely compliant, the skin of the foot sole is deformed well before the passenger’s postural reaction to the driver’s manoeuvres. This skin deformation is due to the mechanical interaction (e.g., shear forces) generated by the surfaces in contact (i.e., the skin of the feet and the supporting surface) and the gravity force acting on the body mass (i.e., body weight). Although the shear forces are small relative to the weight force during natural quiet standing, they are readily detectable by the tactile receptors^1^. These shear forces, and the consequent skin transient deformations, activate the mechanoreceptors embedded in the skin allowing the brain to identify the direction and amplitude of the perturbation, before being detected by other sensory inputs^2^ (e.g. visual, vestibular, or proprioceptive). These tactile inputs contribute to shaping the postural responses during balance perturbations according to the identified limits of postural stability^3,4^. Being the first receptors to be stimulated by the relative motion of the foot and the supporting surface, plantar tactile receptors provide crucial information for controlling balance. Their importance is notably evidenced by the impaired balance control of animals (e.g., cats) and humans after a loss a foot cutaneous feedback^5–7^. Further evidence for the relevance of plantar sensory feedback comes from a study showing that blindfolded human participants are able to scale their postural adjustments according to near-threshold tactile stimulation evoked by small lateral acceleration (~ 0.2 m/s^2^) of the supporting surface before mediation of vestibular or proprioceptive feedback^2^. Interestingly, Fabre and colleagues^8^ recently reported in patients with bilateral vestibular loss that during quiet standing the response of the sensorimotor cortex to foot cutaneous stimulations (light electrical stimulation) was much greater than in age-matched healthy participants.

The low perceptual threshold of tactile receptors relative to others (e.g., vestibular or proprioceptive) for detecting a relative horizontal acceleration of the support surface^2^ suggests a high responsiveness of the tactile sensory system. This responsiveness could have a twofold origin. First, it could stem from the richness of the receptor types (fast, and slow adaptive, FA and SA, type I or II for the main tactile receptors) and from the characteristics of the receptors’ receptive fields. These units have receptive fields round or oval in shape, extended or small, with sharp or blurred boundaries, with the point or points of highest sensitivity eccentrically located within their receptive fields^9^. Secondly, it could also stem from the great compliance (i.e., deformation) of the skin in which the receptors are embedded, which depends, in part, on the footprint (epidermal ridges) and their orientation. For instance it has been showed that the skin vibrations sought to stimulates the Pacinian tactile receptors, were amplified when using a fake finger with the main geometrical characteristics of the human fingertip than with a smooth finger design (i.e., no ridges)^10^. Besides, this amplification is all the more enhanced when the ridges of the fingerprints are oriented perpendicular to the scanning direction^11^.

While the interaction between a surface and the skin has been extensively studied during finger exploration of a surface^12,13^, intriguingly, most of the investigations in the field of balance control have ignored the surface/body contact mechanics. This is particularly surprising given that the foot sole shows footprints (dermatoglyphs) that have similar types and density of forms as fingerprints (e.g., ~ 60% of loops are shared by fingerprint and footprints^14^). Skin deformation during tactile exploration depends not only on the morphological, topographical, and mechanical properties of the skin as mentioned above, but also on the properties of the surface in contact with the skin (i.e., materials, topographic features such as amplitude differences, adhesion, spatial frequency^15^). In this light, a biomimetic surface, whose texture is inspired by the mechanoreceptors and footprint spatial characteristics should optimise skin deformation and are likely to facilitate the neural encoding of this deformation together with the cortical sensory processes of the tactile inputs. Here, to specifically test this hypothesis while maintaining an upright position, we recorded and compared the amplitude of the cortical response (i.e., P_1_N_1_ somatosensory evoked potential, SEP) evoked by the motion of the surface on which the participants were standing. Because it represents the amount of sensory input processing at the cortical level^16–18^ the amplitude of the P_1_N_1_ SEP component was expected to be greater when the participants stood on a biomimetic surface than on other control surfaces (e.g., smooth or grooved) *(Experiment* 1).

Moreover, since an efficient sensory processing allows a better detection of threats to balance, the use of a biomimetic surface should decrease the cognitive demand associated with maintaining equilibrium during the motion of the support surface. To test this hypothesis, we compared the changes of theta band power (4–7 Hz) evoked by the motion of the biomimetic and control surfaces. Indeed, recent studies have shown that an increased theta power over sensorimotor areas is an electrophysiological biomarker of the increased difficulty of the balance task. For instance, a significant increases of theta power in the left sensorimotor cortex before imminent rightward or leftward loss of balance was found^19^. This localized change of theta power spreads afterwards over other cortical areas (e.g., anterior parietal and anterior cingulate areas). A similar increase of theta band activity was observed during the preparation of a challenging balance recovery task which required participants to keep the feet in place and to refrain from stepping responses^20^. Increased theta band activity is also observed in the posterior parietal cortex (PPC) during the transition from a stable to an unstable surface^21,22^. This is in line with the responses of the superior PPC to tactile stimulation (in Monkey) which occurred ~ 60 ms following SI responses^23^. Since the PPC is involved in sensory information integration and generates decision-related activity^24^, this associative cortical area is likely to be involved in the motor recovery response to balance perturbation.

Based on the premise that balance control requires a minimum state of attention and cognitive resources^25^ facilitating the detection of balance instability when standing on a biomimetic moving surface should decrease the attentional demand required for standing steadily. By using a dual task (DT) paradigm (*Experiment* 2) in which participants are involved in a high demand cognitive task (identifying numbers), we expected less interference between the postural and cognitive tasks when participants stood on a biomimetic surface compared to other surfaces (e.g., grooved). This should result in a better performance in the cognitive task or a sharper postural reaction to the surface motion (i.e., large, short-duration postural reactions^26^).

## Experiment 1

### Results

#### Augmented peripheral stimulation by the biomimetic design of the surface

**Figure 1:**
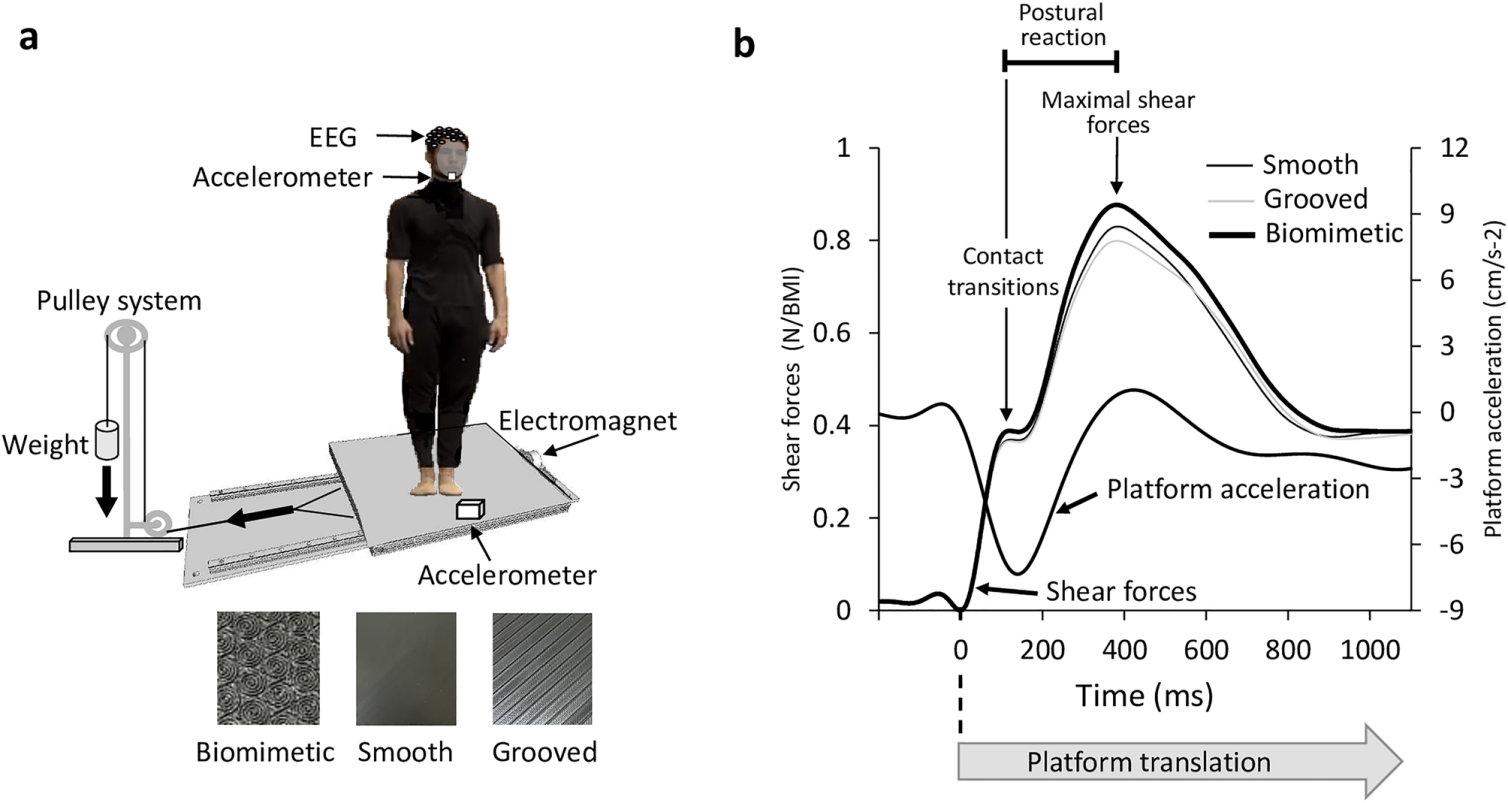
**(a)** Experimental set up. The participant stood on one of the three surfaces glued onto the force platform which, on deactivation of the electromagnet, would undergo a translation due to gravity loading. They wore a safety harness (not shown in the figure) attached to the ceiling (not shown). (**b**) Mean lateral forces and platform acceleration of the 15 participants. At the platform translation onset (broken line) two consecutive phases in lateral force were identified. The first peak force (here smoothed due to the average) corresponds to the maximal extensibility of the skin under the feet until the frictional force (i.e., shear force) cannot anymore resist the sliding leading to transient variations of the local strain distribution (“skin-surface contact transitions”). Afterwards, the force increased and corresponds to a postural reaction.

**Figure 2:**
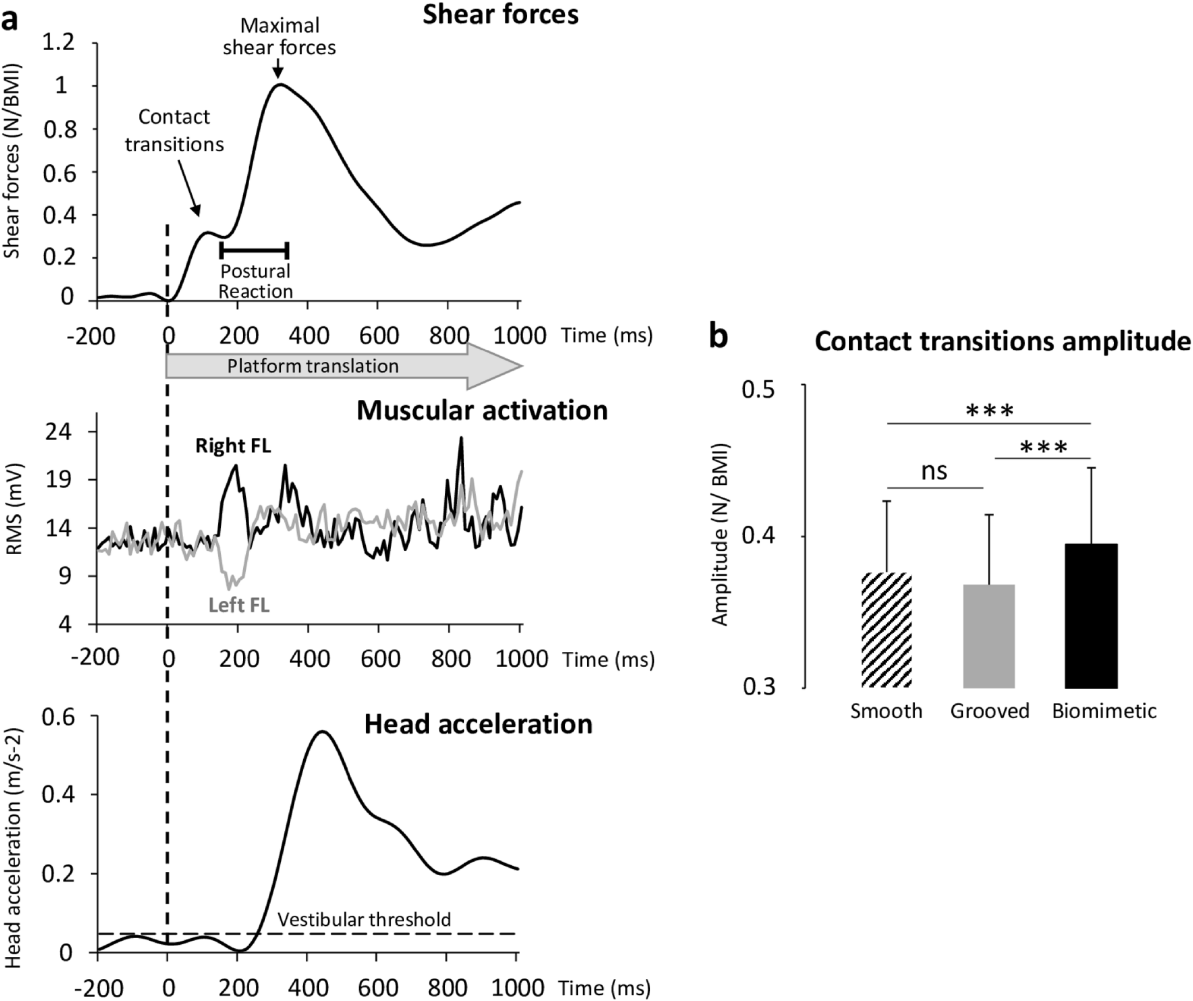
(**a**) Shear forces (top panel), muscular activities (middle panel) and head acceleration (bottom panel), of all trials of one typical participant standing on the smooth surface. The time 0 (broken line) corresponds to the onset of the platform translation. (**b**) Mean contact transitions Force amplitudes /BMI for all 15 participants computed on the three surfaces (smooth, grooved, biomimetic). Error bars represent standard deviation across participants; ***p < 0.001, *ns* not significant. Note that the 1000 ms time window encompasses both the somatosensory-evoked potential and the postural reaction in response to the tactile stimulation (i.e., the rightward platform motion).

As shown in Fig. 1, the shear forces increased in the leftward direction at the onset of the rightward platform motion until a clear break down point was reached (i.e., contact transitions, see methods). The amplitude of this early force differed significantly between surfaces (i.e., Contact transitions amplitude, Fig. 2b; F_2,28_ = 13.84, p = 6.6 × 10^–5^; η^2^ = 0.49). Post-hoc analyses revealed that the force amplitude was greater for the biomimetic (0.40 ± 0.05 Newton normalized to the body mass index considering both the weight and the height of each participant, N/BMI) than for the smooth and grooved surfaces (p = 9.4 × 10^–4^ and p = 1.18 × 10^–4^, respectively), which did not differ significantly (p = 0.20; overall mean of 0.38 ± 0.05 N/BMI). The latency to reach the break down point did not depend on the surface (overall mean of 117 ± 12 ms; F_2,28_ = 0.12, p = 0.89). It occurred a few milliseconds (−26 ± 11 ms) before reaching the maximal value of the platform acceleration for all surfaces (no significant surface effect F_2,28_ = 0.82, p = 0.45). The maximal amplitude reached by the platform acceleration did not differ significantly between surfaces (F_2,28_ = 1.54, p = 0.23).

In addition, the EMG analyses (Fig. 2a) showed that the activity of the right long fibular muscle (FL muscle, acting in the lateral direction to stabilize the ankle joint) started to increase 40 ± 20 ms after the break down point and the latencies of muscular activation were not significantly affected by the surface (F_2,24_ = 0.64; p = 0.54). The left FL activity decreased simultaneously with the right FL activation in 10 out to 13 participants. The delay of the changes in the FL muscles activities relative to the break down point suggested that the early shear forces were not muscularly induced, but rather passively evoked by the friction between the stretched skin and the platform. Besides, the fact that the muscular synergy (right FL activation/left FL inhibition) was initiated after the “skin-surface contact transitions” (i.e., during the postural reaction, Fig. 2a) suggested that it was engaged in the generation of the postural reaction.

During muscle activities, the shear forces continued to increase until a second peak was reached before reducing the forces. This second increase considered as a postural reaction^27,28^ was not altered by the surface textures, neither with respect to its amplitude (F_2,28_ = 2.50; p = 0.10; η^2^ = 0.15, mean = 0.53 ± 0.10 N/BMI) nor its duration (F_2,28_ = 0.20; p = 0.82; η^2^ = 0.014, mean = 296 ± 64 ms). During the postural reaction, the head started moving (i.e., accelerate) with a latency of 172 ± 38 ms relative to the platform motion onset (Fig. 2a). This lag, which likely resulted from the body mass inertia, was not significantly affected by the surface texture (F_2.28_ = 1.48; p = 0.25).

### Cortical facilitation of sensory process when standing on a biomimetic surface

To determine whether the SEP (i.e., P_1_N_1_) originated from tactile and/or from vestibular peripheral inputs, we compared the latencies of P_1_ and N_1_ relative to the vestibular stimulation onset. The vestibular stimulation threshold was defined as the first instance head acceleration exceeded 0.048 m s^−2^ (i.e., threshold for vestibular stimulation^29^). This latency was not significantly affected by the surface texture (F_2,28_ = 1.75; p = 0.19). Paired t-tests showed that P_1_ and N_1_ latencies significantly preceded vestibular stimulation onset for all the surfaces (see Table 1). This indicated that the SEP was not evoked by vestibular inputs, but more likely by tactile inputs originated from the early shear forces (i.e., skin strain) evoked by the platform motion.

**Table 1:** Mean latencies of all participants (n = 15) and inter participant standard deviation (SD) for the P1, N1 and the time when the head reached the vestibular threshold as a function of the surfaces on which participants were standing.

Smooth		Grooved		Biomimetic	
Vestibular threshold					
211 ms (± 40)		225 ms (± 27)		207 ms (± 32)	

P1	N1	P1	N1	P1	N1
138 ms (± 11)	186 ms (± 15)	137 ms (± 13)	184 ms (± 18)	128 ms (± 10)	187 ms (± 13)

T test					
t = 7.90; p < 0.001	t = 2.79; p = 0.01	t = 12.12; p < 0.001	t = 5.50; p < 0.001	t = 10.26; p < 0.001	t = 2.68; p = 0.02

The paired t test corresponds to the comparison between the P1 or N1 and the vestibular threshold.

**Figure 3:**
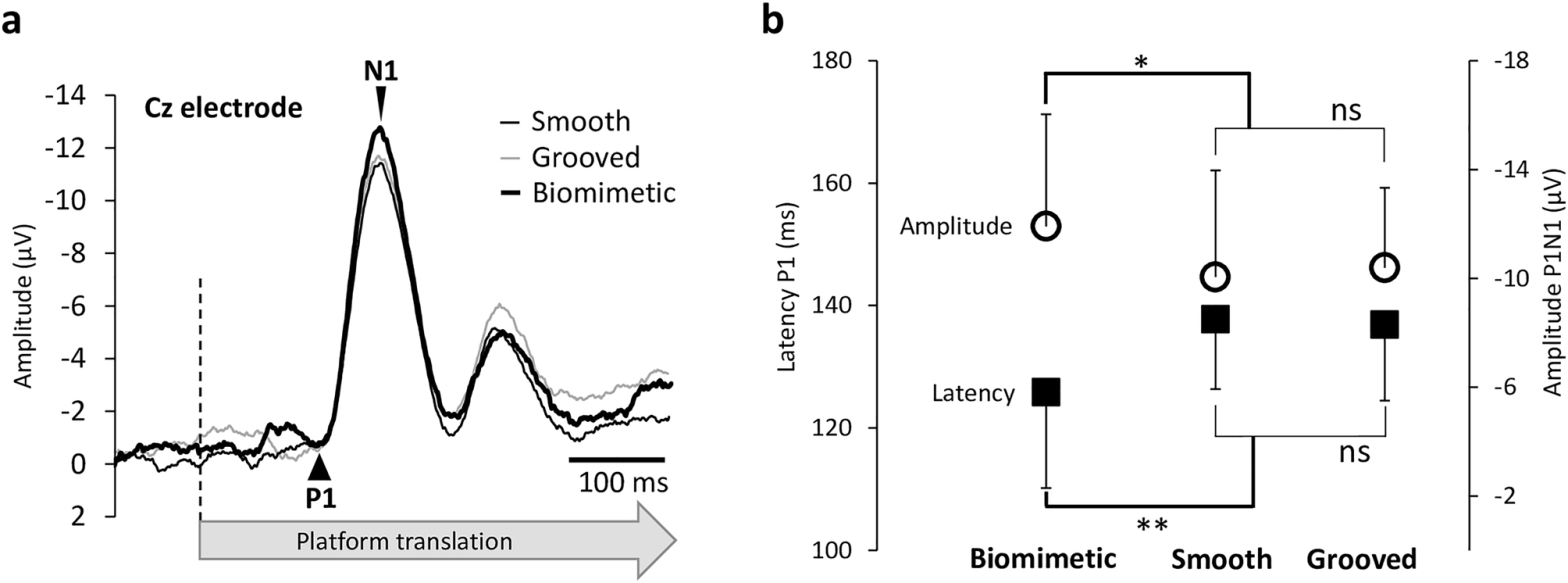
(**a**) Grand average (n = 15) of the SEP recorded over Cz electrode for the 3 surfaces (biomimetic, smooth, grooved). The broken line indicates the start of the stimulation (i.e., translation onset). (**b**) Mean P1 latency and amplitude of the averaged P1N1 SEP for all participants on the three surfaces (biomimetic, smooth, grooved). Error bars represent standard deviation across participants, *p < 0.05; **p < 0.01; *ns* not significant.

Importantly, the latency of P_1_ was shorter and the amplitude of the P_1_N_1_ greater for the biomimetic surface than for the smooth and grooved surfaces (Fig. 3, F2.28 = 8.06, p = 0.002; η^2^ = 0.37 and F_2,28_ = 3.56, p = 0.04; η^2^ = 0.20, for latency and amplitude respectively). The P_1_ latency and P_1_N_1_ amplitude did not differ between the control surfaces (p = 0.75 and p = 0.65, for latency and amplitude respectively). It is worth noting that P1 occurred on average 11 ± 10 ms after the early peak force was reached for the biomimetic surface. The P_1_ short lag relative to the peak force suggests that the SEP is not evoked by the behavioral change (i.e., greater amplitude of the shear forces) when standing on the biomimetic surface. This is confirmed by the t-test of means against a reference value of 40 ms (t_14_ = −10.40, p = 5.76 × 10^–8^), considered as the minimum lag required for a tactile stimulus to evoke a cortical response^30^. Rather this suggests that P_1_ was evoked by tactile afferent inflow occurring before the peak was reached.

To verify if the SEP facilitation (i.e., shorter P_1_ latency and greater P_1_N_1_ amplitude) observed with the biomimetic surface could be linked to a change in leg muscle activity, we compared the general muscle activation (i.e., iEMG, see methods) of the right and left FL during the N_1_ latency interval (i.e., from the onset of the surface motion). The ANOVA did not show a surface texture effect on the iEMG (F_2.24_ = 0.57; p = 0.57 and F_2.24_ = 1.11; p = 0.34, for the right and left FL, respectively). These results suggest that the changes in the SEP observed over the somatosensory cortex when the participants stood on the biomimetic surface stemmed from an increased afferent volley from the foot sole mechanoreceptors rather than from an altered motor command (i.e., muscle activity).

**Figure 4:**
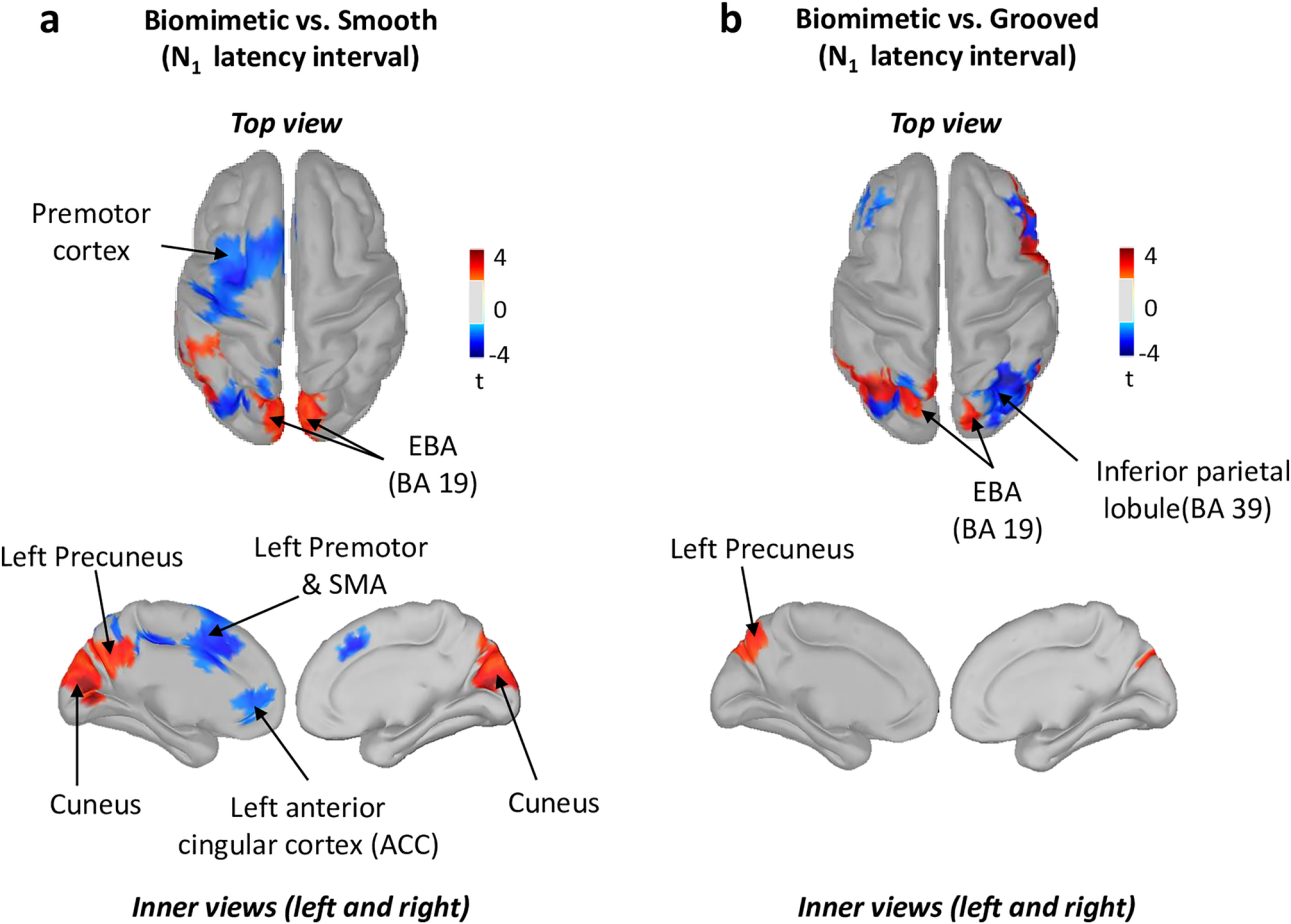
Statistical source estimation maps for biomimetic versus smooth (**a**), biomimetic versus grooved (**b**) contrasts. Significant t-values (p ≤ 0.05, n = 15) of the source localization were shown during the time window from 0 ms to N1 latency. Sources are projected on a cortical template (MNI’s Colin 27). For each contrast, we display the top, and the left and right inner cortical views.

Neural sources of the SEPs (see methods for their computation) revealed significant greater activation in the left precuneus (medial extent of Brodmann area 7 of the PPC, Fig. 4 warm colour) for the biomimetic surface for both the biomimetic/smooth (Fig. 4a) and biomimetic/grooved (Fig. 4b) contrasts. The same contrasts also showed significantly greater activity of the extrastriate body areas (EBA, BA19). On the other hand, these contrasts revealed greater activities of the left premotor (PM) and anterior cingular (ACC) cortices (see cold colors in Fig. 4a) for the smooth surface and greater activation of the right inferior PPC (BA39) for the grooved surface (Fig. 4b, cold colour).

### Modulation of theta (5–7 Hz) oscillations

**Figure 5:**
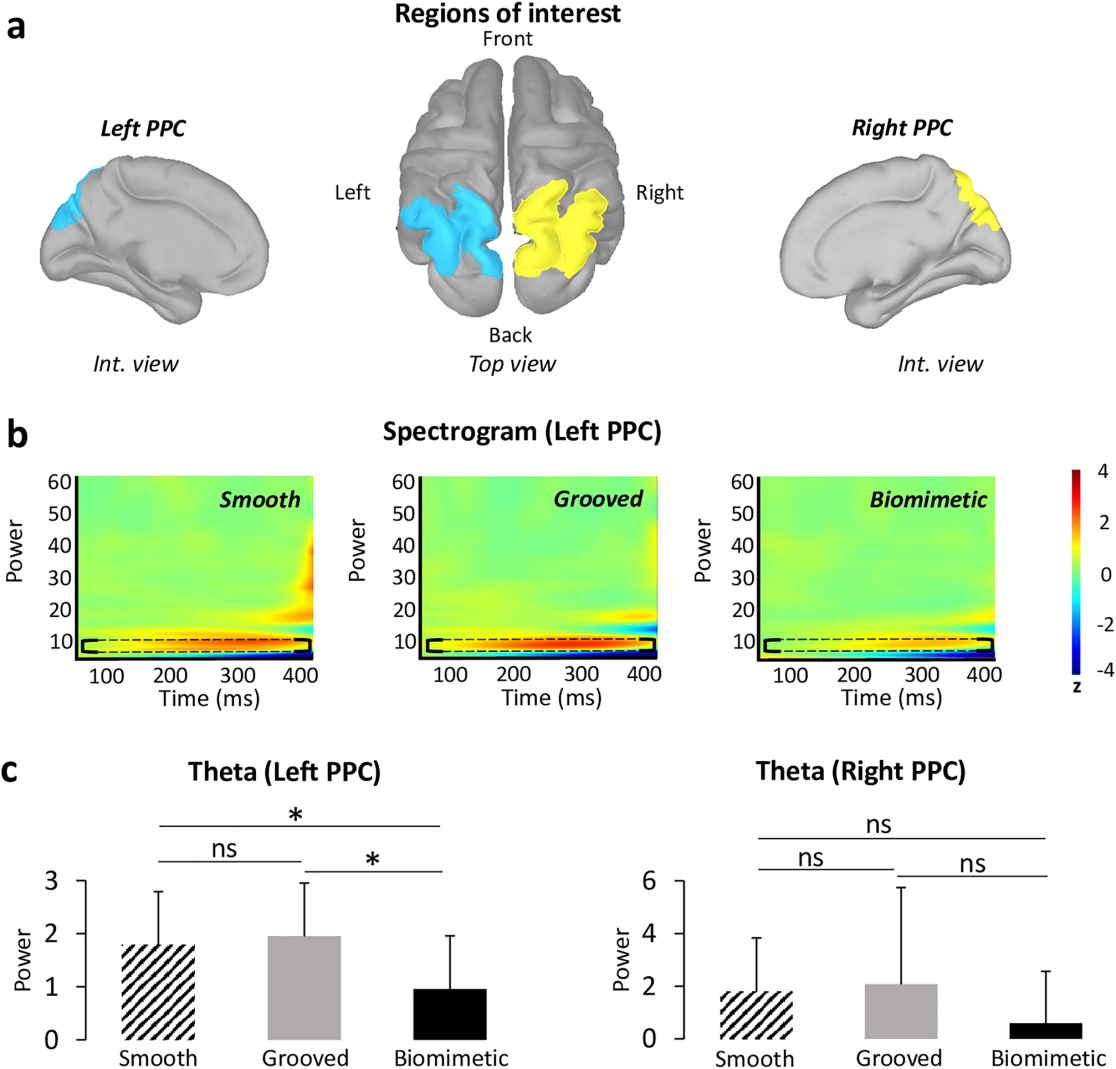
(**a**) Localization of the regions of interest (ROIs) on the anatomical MRI Colin 27 brain template that was used to compute cortical activations. Note that similar ROIs were defined for the left and right parietal posterior cortex (PPC). (**b**) Time–frequency power (ERS/ERD) of the signals by means of a complex Morlet’s wavelet transform applied on the ROIs for each surface of each participant then averaged. Cooler colors indicate ERD and warmer colors, indicates ERS. Frequency bands from 1 to 60 Hz were provided to have an overview of the full spectral content of cortical neural oscillations. We have shown the spectrum from 0 to 400 ms to focus on the analyzed time window of the ERS/ERD (thereby removing edge effects). The theta band has been circled by a black dotted line for each surface. (**c**) Mean of theta (5–7 Hz) frequency band computed during (0; 400 ms) time window in the left and right PPC for each surfaces (smooth, grooved, biomimetic). Error bars are standard error across participants, * p < 0.05, *ns* not significant.

The time–frequency analysis was computed over the first 400 ms of the platform translation (which included the P1N1 SEP) in the PPC because this region activity is considered as a neural signature of imminent loss of balance (Fig. 5a). The results showed that theta power specifically in the left PPC (Fig. 5b) was significantly modulated by the type of surface on which the participants were standing (F_2.28_ = 3.99; p = 0.03). Post-hoc analyses revealed that it was significantly smaller for the biomimetic than for the smooth and grooved surfaces (p = 0.04 and p = 0.03, respectively), with no significant difference between the two control surfaces (p = 0.67) (Fig. 5c). This effect was lateralized to the left hemisphere as it was not observed in the right PPC (F_2.28_ = 1.32; p = 0.28) (Fig. 5c).

### Results of experiment 2

**Figure 6:**
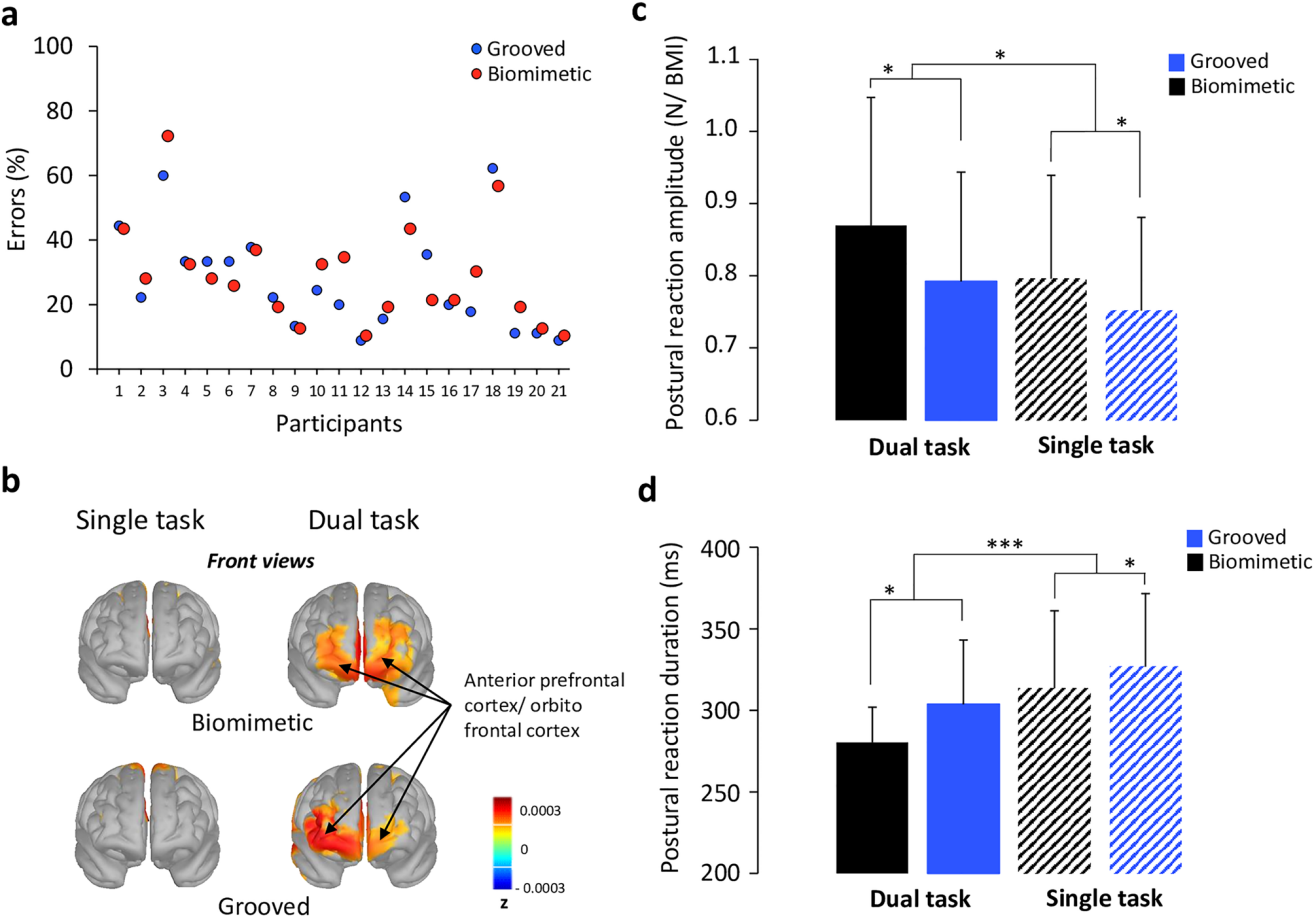
(**a**) Percentage of errors for the cognitive task of each participant on both surfaces (grooved, biomimetic). (**b**) Source localization during the time window from -2000 to 0 ms latency interval on both surfaces (grooved, biomimetic) during solely the motor task (i.e., single task) and the dual task. We display the front view of the sources projected on a cortical template (MNI’s Colin 27). (**c**) Mean postural reaction amplitude normalized by the BMI for all participants computed on both surfaces (grooved, biomimetic) during solely the motor task (i.e., single task) and the dual task. Error bars represent standard deviation across participants; *p  <  0.05) (**d**) Mean postural reaction duration for all participants (n  =  21) computed on both surfaces (grooved, biomimetic) during solely the motor task (i.e., single task) and the dual task. Error bars represent standard deviation across participants; *** p  <  0.001).

To identify the magnitude of the interference in the dual task paradigm we analysed cognitive and balance-related motor task performances. For the cognitive task, participants were instructed to silently count the number of times that 7 was part of a series of ten different three-digit numbers that were spelled out at high speed and an error was counted each time that the participant reported an incorrect number of occurrences of the number 7. A paired t-test did not reveal significant difference in the percentage of errors between the biomimetic and the grooved surfaces (t_20_ = −0.20; p = 0.85; 24 ± 11.5%). As shown in Fig. 6a, 2 out of 21 participants exhibited values three times above the standard deviation of the mean (i.e., 59% and 66% of errors). These large errors suggest that the task was too difficult for these participants or that they did not allocate enough attentional resources to the cognitive task. These participants were excluded from the analyses. Furthermore, the activity observed over both the anterior prefrontal and orbito-frontal cortices during the last part of the counting (i.e., over a 2000 ms interval before the platform motion) was greater in the dual task than in the single task (Fig. 6b). This confirmed the participants' engagement in the cognitive task^31^. Note that no such activities of the frontal lobe were observed in the 2 participants that were discarded from the analyses, due to their high error rate.

For the balance-related motor task, the ANOVA indicated that the maximal force during the postural reaction was of greater magnitude when performing the dual task (combined cognitive and motor tasks) than the single motor task (F_1,18_ = 4.78; p = 0.04; η^2^ = 0.21), and when standing on the biomimetic as compared to the grooved surface (F_1,18_ = 5.92, p = 0.03; η^2^ = 0.25) (Fig. 6c). No significant interaction was observed between Task and Surface texture (F_1,18_ = 0.76; p = 0.39; η^2^ = 0.04). The ANOVA also revealed that the duration of the postural reaction was shorter when performing a dual task (F_1,18_ = 21.18; p = 2.21 × 10^–4^; η^2^ = 0.54) and when standing on the biomimetic surface (F_1,18_ = 6.95; p = 0.02; η^2^ = 0.28) (Fig. 6d). The interaction Task × Surface texture was not significant (F_1,18_ = 0.43; p = 0.51; η^2^ = 0.02). Although the ANOVAs performed on the variables relating to the postural reaction did not reveal significant Task × Surface texture interactions, the results of planned contrasts post-hoc tests revealed that the postural reaction was of greater amplitude (F_1,18_ = 10.51; p = 4.52 × 10^–3^) and smaller duration (F_1,18_ = 37.70; p = 8 × 10^–6^) when standing on a biomimetic surface and performing the dual task than the in the other conditions (Fig. 6). This suggest greater efficiency of the postural reaction when participants stood on the biomimetic surface in the dual-task condition (i.e., greater amplitude, Fig. 6c and smaller duration, Fig. 6d of the postural reaction).

## Discussion

Complying with both dermatoglyphs and mechanoreceptors characteristics, the biomimetic surface in contact with the feet evoked faster and greater cortical response to the platform lateral motion (i.e., P_1_N_1_), with respect to both control surfaces (i.e., smooth and grooved). The greater amplitude of the SEP, when standing on the biomimetic surface, suggests augmented cutaneous afferent processes^16–18^. The increase P_1_N_1_ amplitude and the shortening of the evoked response latency (i.e., P_1_) could be due to an accentuation of the deformation of the compliant skin when interacting with the biomimetic surface. One could argue that the increase P_1_N_1_ amplitude and the shortening of the evoked response latency (i.e., P_1_) could be due to the increase amplitude of the peak force. However, the latency of P_1_ relative to this peak (about ~ 10 ms) suggests that the inputs come from the skin/surface contact before the peak of the transient skin deformation was reached. This reduces the possibility for a significant role of the peak force in generating the SEP. Nevertheless, the larger peak amplitude represents the enhanced skin deformations while no concomitant change of its duration was observed. This is in line with a study showing that a greater deformation of the fingers’ skin generates greater friction force, and stress that induce stronger tactile stimulation of the mechanoreceptors^32^. Similarly, the interaction of the foot and the biomimetic surface increased the intensity of the skin mechanoreceptors stimulation, which in turn boosted the transmission of tactile signals to the primary somatosensory cortex (SI) where neurons respond to various tactile stimulations^33,34^.

The source analyses showed that changing the standing surface texture significantly altered the current in the left PPC but had no significant effect on the current recorded in SI. This suggests that the sensory facilitation observed with the biomimetic surface may have involved direct thalamocortical projections to the PPC. The basis for this suggestion is twofold. First, neuroanatomical studies in the macaque have shown direct projections of cutaneous information from the thalamus to the PPC^35,36^. Secondly, our results showed an increased activation of the medial extent of the SPL (i.e., precuneus) for the biomimetic surface, with respect to both control surfaces, in line with functional interactions (e.g., somatomotor function) between the precuneus and the thalamus^36,37^. In this light, the shared connectivity between the precuneus and the extrastriate body area (EBA)^38^, which showed an enhanced activity with the biomimetic surface texture, is consistent with the role of the EBA in enhancing the local spatial processing of body information on the direction of a stimuli out of view^39^, such as the skin stretch under the feet in the current study.

The shorter latencies of the SEP observed when the participants stood on the moving biomimetic surface could suggest some contribution of muscle-stretch receptors, as it is well-established that muscle spindle endings are extremely sensitive stretch receptors ^40,41^. However, the evoked potentials that arise from the stimulation of the lower limb nerve endings exhibit much shorter latencies (i.e., 20–65 ms^40,41^) than those observed in the current study (126 ms, for the biomimetic surface). Furthermore, the increased activity of the leg muscles induced by the translation of the biomimetic surface occurred ~ 160 ms after the onset of the translation, i.e., after the P_1_ occurrence. This reduces the possibility for a significant role of muscle-stretch receptors in generating the SEP when the participants stood on the translating surface, irrespectively of the supporting surface texture. Vestibular input is also an unlikely candidate for evoking the cortical response, as P_1_ and N_1_ had shorter latencies than the latency with which the head reached the acceleration threshold for activating vestibular receptors (i.e., 207 ms, for the biomimetic surface).

Finally, the results of theta oscillations analyses were consistent with the likely role of the left PPC in attentional processes^21,22^. Our participants showed a significant decrease in the power of theta oscillations in the left PPC when they stood on the biomimetic surface compared to the control surfaces (i.e., smooth and grooved). Since theta oscillations are considered as a neural correlate of a need for attentional demand in challenging balance tasks (i.e., theta power increases with increased attentional demand^19,21^, the significant decrease of theta-band power with the biomimetic surface may reflect a decrease in the attentional demand and a down modulation in the difficulty of the task^42^. Alternatively, the increased theta power, observed when standing on both control surfaces, may witness the increase attentional and cognitive demands. This is consistent with the greater activities observed within the pre-motor cortex (e.g., SMA) and ACC of the left hemisphere observed when the tactile salience of the surface decreases, as when standing on a smooth surface (as compared to either the biomimetic or grooved surfaces). Previous studies have suggested that the SMA plays an important role in the control of demanding balance tasks^43^. This was notably evidenced by the significant structural and functional adaptation of the SMA activity after balance training^43^. Therefore, the enhanced activity of the SMA, found when individuals stood on the smooth surface (i.e., with less friction making slipping more likely) may suggest that the standing task was more demanding with this surface than with textured surfaces (biomimetic or grooved). The increased activity observed in the ACC would also be consistent with this suggestion as the role of this cortical region is well-recognized when individuals are uncertain about fulfilling the required task appropriately^44^ or in error-recognition^45^. These interpretations are also supported by the proposed function of the ACC in the regulation of attention and cognitive control^46^. Enhancing the need for attention when standing on a smooth surface could be a mean for withholding potentially erroneous responses due to impoverished tactile cues related to platform motion until other sensory modalities (e.g., vestibular, visual) can resolve the ambiguity of the support displacement. In addition to the increased theta oscillations power for the control grooved surface, the source analyses revealed an increased activation of the right PPC. This findings are consistent with the crucial role of this cortical area in the processing of somaesthetic gravitational information for postural control, as shown in neglect patients after right hemispheric strokes^47^.

Overall, our results point to a reduced difficulty of the balance task when standing on a biomimetic surface. By increasing the attentional load of the balance task, *Experiment 2* confirmed this interpretation. Based on the premise that postural control requires attention^25^, the postural perturbation observed when performing a simultaneous cognitive task would be due to the sharing of limited attentional resources^48,49^. However the intriguing result of *Experiment *2 is that the postural reaction observed during the platform motion rather complied with the spatiotemporal characteristics of an efficient postural reaction in the dual task than in the single task (i.e., shorter duration and greater magnitude^50,51^). These behavioral features were observed to a greater extent when the participants stood on the biomimetic surface. Therefore, the participants’ engagement in the cognitive task did not have a deleterious consequence on the postural control as often reported in previous studies^52,53^. The greatest efficiency of the postural reaction observed in participants standing on the biomimetic surface could stem from a greater capacity to shift the attentional focus from the primary motor task to the secondary cognitive task. Such external focus is known to diminish motor-related conscious attentional processes^42,54^ compared to an internal focus of attention^55^, and improve motor performance. This has been clearly demonstrated in a study in which participants had to make oscillatory movements (ski-type slalom movements) when standing of platform mounted on wheels that ran laterally on two bowed rails^56^. Elastic rubber belts attached to the platform ensured that the platform returned to the center position during the oscillatory movements. The authors showed that the motor performance decreased when the participants’ attention was focused on the force that the feet should exert on the supporting platform (i.e., internal focus) as compared to when their attention was focused on the wheels of the platform (external focus). Overall, the results of *experiment *2*,* combined with those of *Experiment* 1 showing similar postural reactions between the different surfaces, suggest that during small accelerations of the standing platform, the advantage of standing on a biomimetic surface to safeguard stability is solely expressed when one is involved in a dual task (*Experiment* 2). Although it is often the case that one is engaged in a cognitive task while standing (e.g., listening to people, singing while showering, etc.), greater platform accelerations could be needed for the biomimetic surface to express an improvement of the postural reactions compared to other types of surface.

It is possible that the equilibrium demands in response to support motion decreased when standing on a biomimetic surface, as also suggested by the smaller theta power observed in *Experiment* 1 with this surface. The biomimetic surface may therefore facilitate the use of low-level sensorimotor loops, which are less permeable to cognitive load, and which enable speedy performance^54^. As mentioned, thalamic projections to the left pre-cuneus^36,37^, which has dense interconnections with the motor and premotor cortices^57^ may have contributed to the facilitation of the neural responses to the tactile stimulation observed with the biomimetic surface (i.e., increased P1N1 SEP). These thalamocortical connections areas could constitute the neural underpinning of the efficient spatiotemporal pattern of the postural reaction when standing on the biomimetic surface.

A limitation of the present study is that it does not provide information as to whether the behavioral and neurophysiological changes observed when standing barefoot on the biomimetic surface occur when wearing shoes. Even though Chander and colleagues^58^ demonstrated that the barefoot condition exhibited a quicker and more efficient balance response than wearing conventional shoes, the shoe sole material might convey as does barefoot, the friction induced vibration generated by the contacted surfaces. For example, Formula-1 footwear was able to dampen the low frequency (30 Hz) vibration transmitted to the foot and to transfer high-frequency stimuli (200 Hz) into the skin^59^. This particular frequency was reported to be generated by the shear stresses between the foot skin and surface when standing barefoot on the biomimetic surface^60^.

## Methods

### Experiment 1—participants and task

Fifteen participants (9 women) without any known neurological and motor disorders participated in the experiment (mean age 26 ± 3 years, mean weight 64 ± 10 kg). All participants, except two, characterized themselves as right footed. All participants gave their written informed consent to take part in this study, which conformed to the ethical standards set out in the Declaration of Helsinki and all experimental protocols were approved by the STAPS (Science and Technique of Physical and Sports Activities) Research Ethics Committee (CERSTAPS, no. IRB00012476-2021-09-12-140).

Participants were requested to stand barefoot with their feet at a natural distance apart on different types of surfaces (see below), fixed in the middle of a movable force platform. They wore a safety harness attached to the ceiling. We ensured that the feet position remained the same throughout the experimental conditions. As the morphology of the foot (i.e., flat, hollow, standard) can have an impact on body stability^61^, we verified that none of them had any foot morphological particularities. This was done by measuring the width of the forefoot (i.e., metatarsal band from the first to the 5th toe) and the isthmus width localized in the middle of the foot and connecting the forefoot with the rearfoot. Computing the percentage ratio ((isthmus width)/(forefoot width) × 100) allowed us to identify hollow feet (< 33%), standard feet (33% to 50%) and flat feet (> 50%)^61^. All participants showed standard feet, therefore none were excluded from the analyses.

We used a set-up employed in previous studies for stimulating foot tactile afferents^27^. A movable force platform is placed on two parallel rails and is held stationary by an electromagnet (Fig. 1a). A cable is attached to the platform and runs laterally through a pulley system with a load fixed to its extremity. The platform translation is triggered by deactivating the electromagnet. The load is adapted to the weight of the participants, such that switching off the electromagnet allowed the platform to accelerate to the right of the participants, without endangering their balance. A triaxial accelerometer (MEMS, model 4630, Measurement Specialities, USA; 1000 Hz, filtered with a 4th order Butterworth low-pass filter with 10 Hz cut-off frequency) was used to assess the platform acceleration. The peak acceleration reached by the platform did not differ between surfaces (F_2,28_ = 1.54, p = 0.23) and was 8.34 ± 0.74 cm s^−2^. In addition, the latency to reach the peak did not differ between surface (F_2,28_ = 3.27, p = 0.06) and was 196 ± 15 ms. These results suggest that stimulation intensity was similar across all surfaces.

At the start of a trial, the participants looked at a fixation point positioned at eye level, 2 m directly ahead. They were asked to close their eyes upon receiving the verbal information on the nature of the upcoming condition, and to remain still. This information indicated one of these two conditions: platform translation (37 trials) or platform steady (8 trials), which were pseudo-randomly distributed. The later set of trials reduces the possibility of adopting a stereotyped postural set linked to the forthcoming body translations (e.g., slightly leaning). These trials were also used to measure and model the noise contaminating the EEG data (see below). In all trials, the participants had to maintain an upright steady posture during 5 s (i.e., duration of trial recording). The platform translation occurred at any time between 2 and 4 s after providing the information about the platform translation to avoid anticipating the instant of the translation onset. The trials without translation also lasted 5 s. A short break was frequently proposed to the participants during the experiment.

### Surfaces

Participants stood on three different surfaces which were glued onto the platform: a biomimetic surface, a surface with parallel grooves (i.e., no bioinspired features characteristics and a smooth surface (the last two surfaces, grooved and smooth were used as controls). These surfaces were created with a 3D printer (Ultimaker 2+) using biopolymer thermoplastic (Polylactic acid, PLA). Three characteristics were selected to build the biomimetic surface: shape, spatial period, and depth of the ridges.

The biomimetic surface was textured with circular or oval shapes inspired from both the shape of the tactile receptors’ fields that demonstrate a preferential skin strain axis and orientation of this axis, which is not the same for all units^9,62^ and the forms of the dermatoglyphs, which exhibit three main circular forms (loops, whorls, and arcs^63^). We verified whether the radius of curvature of the circular shape of the biomimetic surface complied with the participants’ toe prints. To do so, we used the ink dabbing method to collect the toe prints of each participant on a white sheet of paper. Contrary to fingerprints, toe prints have their characteristic features at the lower end of the phalanges^14^. Then, the rolling of the prints was taken longitudinally from lower end to the upper end of the toe (i.e., opposite direction than when collecting fingerprints). For each participant, we measured, and then averaged, the radius of curvature of the three most visible ridges from three different toes. A t-test of means against a reference value indicated that the radius of curvature on the toe surfaces (4.3 ± 1.1 mm) did not differ significantly compared to the radius of the loops printed of the biomimetic surface (t_13_ = 1, p = 0.34).

The spatial frequency of the biomimetic surface complied with the spatial frequency of the participants’ toeprint ridges. This was confirmed by the result of the t-test of means against a reference value showing that the mean spatial frequency of the biomimetic surface (0.9 mm) was not significantly different from that of the toeprints ridges (0.87 ± 0.06 mm) (t_13_ = −1.66, p = 0.12). Note that the spatial period of the biomimetic surface also corresponded to the distance between the centre of adjacent receptive fields of the mechanoreceptors** (**from 0.9 to 3.8 mm^64^).

Finally, the depth of the valley between the ridges was computed from what we know based on finger surface exploration and balance maintenance literature. The depth to best perceive the stimulus on the finger skin is estimated as 0.1 mm with a 0.5 N normal force^65,66^. In a previous study, we found that the minimum shear forces amplitude to detect support translation beneath the feet standing in a natural position was ~ 3.5 N ^2^. To the condition that normal and shear forces relationship is linear the suggestion is that a 0.7 mm depth of the valley should enable to perceive the minimal shear force when bearing our body weight.

A smooth surface also printed in PLA but without any designed patterns was used as a control surface. While the smooth surface is used as standard control surface, a grooved surface with different texture parameters from the ones of the dermatoglyphs and characterized by the same ridges direction, allows for excluding a simple effect of the local strain variation due to a general texture. The texture of the grooved surface was composed of rectilinear ridges with a depth of 0.3 mm and a spatial frequency of 7 mm. Comparing the biomimetic texture with a grooved texture can then highlight the role played by a geometrical distribution of the texture mimicking the receptive features of the foot skin. Such a biomimetic geometry can give rise to local stress and strain distributions with a specific orientation pattern, which can favour the detection of the transient strain variations by the mechanoreceptor activation.

The order of the three surfaces expositions was counterbalanced across participants. The participants were not informed prior to the experiment about the reason the standing surface was changed during the recording session. The visual appearance of the three surfaces exhibited slight variation, however when the participants were asked after the experiment was completed whether they had perceived that they stood on surfaces having different textures, none of them reported having done so. A reason for the absence of tactile discrimination between the surfaces could be the lack of voluntary motor interaction with the surfaces. Indeed, our haptic sense relies heavily on the active exploratory movements with the object surfaces^13^. During the experiments, we devoted special attention to prevent voluntary exploratory movements of the feet with the surface during the whole experimental session.

### Recordings and analyses

Electroencephalography (EEG) activity was continuously recorded from 64 Ag/AgCl surface electrodes embedded in an elastic cap (BioSemi ActiveTwo system: BioSemi, Netherlands). Specific to the BioSemi system, “ground” electrodes were replaced by Common Mode Sense active and Driven Right Leg passive electrodes. The signals were pre-amplified at the electrode sites, post amplified with DC amplifiers, and digitized at a sampling rate of 1024 Hz (Actiview acquisition program). The signals of each electrode were referenced to the mean signal of all electrodes. Four Ag/AgCl electrodes placed near the canthus of each eye and under/over the left eye orbital allowed us to control for blinks, and horizontal and vertical eye movements.

The continuous EEG signal was segmented into epochs synchronized relative to the onset of the platform translation, which was identified at the onset of the monotonic increase of the shear force. After artefact rejections based on visual inspection, for each participant and surface, a minimum of 96% of the trials were included in the analyses. The signals were filtered off-line with a 50 Hz digital notch filter (24 dB/octave) and with a 0.1–48 Hz band-pass digital filter (48 dB/octave) implemented in BrainVision Analyzer 2 software (Brain Products, Germany). For each participant, the SEPs were obtained by averaging all epochs of each surface for each participant. The average amplitude computed 50 ms prior to the platform translation served as baseline. Consistent with studies recording cortical potentials evoked by lower limb stimulation, the SEPs were found to be maximal over the vertex (Cz electrode)^67^. Therefore, this electrode was used to assess the SEPs. We primarily based our analyses on the P_1_N_1_ wave evoked by the sensory stimulation induced by the platform translation. The amplitude of P_1_N_1_ was measured peak to peak, and its latency was assessed measuring the P_1_ latency.

### Cortical sources

Neural sources of the SEPs were estimated with the dynamical Statistical Parametric Mapping (dSPM^68^) implemented in the Brainstorm software. A boundary element model (BEM) with three realistic layers (scalp, inner skull, and outer skull) was used to compute the forward model on the anatomical MRI brain template from the Montreal Neurological Institute (MNI Colin27). Using a realistic model has been shown to provide more accurate solution than a simple three concentric spheres model^69^. We used of a high number of vertices (i.e., 15,002 vertices) to enhance the spatial resolution of the brain template. Such EEG source reconstruction has proved to be suited to investigating the activity of outer and inner cortical surfaces with 64 sensors^70^. Measuring and modelling the noise contaminating the EEG data is beneficial to source estimation. Noise covariance matrices were computed using the eight trials with the platform steady condition, while the participants stood still. The current maps were averaged from the start of the shear forces to N1, for each participant and surface texture.

The data were transformed into time–frequency domain using Morlet wavelet transforms. We used a 1 Hz central frequency (full width at half maximum FWHM tc = 3 s) which offers a good compromise between temporal and spectral resolutions^71^. The power of theta (5–7 Hz) was computed for each trial in the source space in a region of interest (ROI, 589 vertices) encompassing the left inferior and superior PPC (based on the Destrieux cortical atlas^72^). Then, the signal was expressed as a change of theta power computed over the first 400 ms of the platform translation (which included the P1N1 SEP) with respect to a 350 ms window baseline taken before the translation (−400 to −50 ms). For each participant, the resulting event-related synchronization/desynchronization (ERS/ERD) was then averaged across trials and surface textures. The same procedure was applied with the signals computed in a control ROI (650 vertices) encompassing the inferior and superior PPC of the right hemisphere.

### Behavioural recordings and analyses

The ground reaction forces and moments were recorded with an AMTI force platform (60 × 120 cm, Advanced Mechanical Technology Inc., USA) at a sampling rate of 1000 Hz (filtered with a 4th order Butterworth low-pass filter with 10 Hz cut-off frequency). The analysis focused on the shear forces along the mediolateral (M/L) axis. M/L shear forces are horizontal forces that act in parallel to the contact surface, resulting in the relative sliding or friction between contacting surfaces (i.e., foot skin-surface interaction). The onset of the shear forces provided a reliable temporal landmark to determine the onset of the cutaneous stimulation evoked by the platform translation. The onset of this stimulation was defined as the first instant the M/L shear forces started to increase monotonically. Figure 1b shows the dynamics of the M/L shear forces resulting from the platform acceleration.

A general pattern of events emerged:

(i) The first phase shows a ramp of the platform acceleration (constant jerk) corresponding to a ramp in the traction force; this phase lasted on average 196 ± 15 ms leading to a platform displacement of about 0.54 ± 0.05 mm. Due to the inertia of the body, this displacement is accommodated mainly by the skin deformation. The peak amplitude of this force was measured with respect to its baseline value (i.e., computed during a period of 200 ms prior to the translation onset).
(ii) In a following transition phase the jerk decreases and eventually becomes negative. This is a crucial phase, where the contact between the skin and the surface is characterized by high superficial shearing, leading to transient variations of the local strain distribution (“*skin-surface contact transitions*”), which are directly affected by the topography of both the skin and the surface itself; moreover, local detachments and slipping can occur, leading to transient deformations and waves propagating in the skin, which are likely to activate the mechanoreceptors (“*contact stimuli*”); a similar phenomenon has been observed in literature, when considering the motion of a surface under a stationary fingertip, showing that the deformation of the skin increases until the frictional force (i.e., shear force) cannot anymore resist the sliding (i.e., stick/slip phenomenon^73^); within this phase, the shear forces result from a balance between inertia effects and contact accommodation.
(iii) Afterwards the shear forces continued to increase until a second peak was reached before decreasing. This second increase can be considered as predominantly a postural reaction^27,28^. The duration of the postural reaction was defined as the time elapsed between these peaks.

The amplitudes of both peaks of the shear forces were normalized to the body mass index (BMI) of each participant.

Head acceleration was measured with a triaxial accelerometer (model 4630, Measurement Specialities, USA; 1000 Hz, filtered with a 2nd order Butterworth low-pass filter with 10 Hz cut-off frequency) placed on the participants’ chin. We measured head acceleration to evaluate the latency of the vestibular stimulation induced by the platform translation. The onset of the vestibular stimulation was defined at the first instant head acceleration exceeded 0.048 m s^−2^ (i.e., threshold for vestibular stimulation^29^). We measured the lag between this threshold and shear forces onset to determine if the vestibular stimulation occurred after the stimulation of the foot mechanoreceptors.

Bipolar surface electromyography (EMG, Bortec AMT-8 system, Bortec Bomedical, Canada) was used to record the activity of the long fibular muscle (FL) of both legs. The FL muscles are responsible (with other muscles) for controlling stance. They contribute to the eversion movement of the foot, and also to the maintenance of the arch of the foot to ensure optimal postural stability^74^. The FL EMG signals were pre-amplified at the skin site (1000×), sampled at 1000 Hz, band pass filtered from 20 to 250 Hz, and rectified. Two participants were excluded from the EMG analyses due to noisy EMG signals. To quantify the muscle activity, we computed the integral of the EMG activity (iEMG) over two intervals. The first corresponded to the “resting interval”. It lasted 1 s and ended at the onset of the platform translation. The second interval covered the time elapsed between the platform onset and the N1 component of the SEP. The duration of this second period was specific to each participant. In order to be able to compare muscle activities between the two intervals, we normalized, for each participant, the EMG activity of the resting interval prior translation onset, to the duration of the second interval “N1 latency” (~ 180 ms). We also calculated the latencies of EMG changes relative to the onset of the platform translation. This was done by first computing the mean and standard deviation of the muscle background activity (i.e., during the resting interval) for each participant and surface. The onsets of the changes in EMG activity were defined as the instant at which the EMG activity increased above or decreased below a threshold level set at twice the standard deviation of the mean background activity.

### Experiment 2—participants and task

The goal of *Experiment* 2 was to test whether the attentional demand required for equilibrium maintenance is reduced when standing on the biomimetic surface, compared to a control surface. To further compare the effect between standing on a biomimetic and standing on a non-biomimetic textured surfaces, the grooved surface used in *Experiment* 1 was selected as the control surface. The choice of the grooved surface was motivated by the fact that its comparison with the biomimetic surface allows for excluding a simple effect of the presence of texture on the surface and can then highlight the role played by a geometrical distribution of the texture mimicking the receptive features of the foot skin.

Twenty-one new participants (7 women) without any known neurological and motor disorders participated in the experiment (mean age 22 ± 2 years, mean weight 67 ± 10 kg). All participants gave their written informed consent to take part in this study, which conformed to the ethical standards set out in the Declaration of Helsinki and all experimental protocols were approved by the STAPS (Science and Technique of Physical and Sports Activities) Research Ethics Committee (CERSTAPS, no. IRB00012476-2021-09-12-140).

The procedure was identical to that in *Experiment* 1 with one exception that pertained to the dual task (DT) paradigm. We used a demanding cognitive task to increase the participants’ attentional load while their supporting surface was translated as in *Experiment* 1. Participants were asked to listen to a series of ten different three-digit numbers spelled out at high speed (presented orally by a computer at a frequency of 1 Hz) which ended at any time before the platform translation. The series of numbers varied across trials but were the same for both surface textures and participants. Participants were instructed to silently count the number of times that 7 was part of the three-digit numbers and to provide their response at an auditory tone occurring 3 s after the platform translation onset (i.e., well after the data analysed intervals). The same procedure was used for the trials in the steady platform condition. We counterbalanced the presentation of the different conditions (i.e., biomimetic or grooved surfaces, with or without translation; single or dual tasks) across participants but prevented the occurrence of two successive textures involving the dual task for fatigue prevention.

The participants’ performance in the cognitive task was assessed by computing, for each surface texture, the average percentage of errors ((number of errors/10 numbers) × 100). An error was counted each time that the participant reported an incorrect number of occurrences of the number 7.

### Statistical analyses

The behavioural and EEG data were submitted to separate analysis of variance (ANOVA) with repeated measurements. In *Experiment 1*, one-way ANOVAs were used for mean comparisons with the support surface (Smooth, Grooved, Biomimetic) as the intra-participants factor. We computed statistical maps by contrasting the current maps (i.e., each vertex) computed when standing on a biomimetic surface and control surfaces using t-tests (significance threshold p < 0.05)^75^. We applied an FDR (False Discovery Rate) correction for multiple comparisons across regions^76^.

In *Experiment* 2, a 2 × 2 ANOVA was used for mean comparisons with the support surface (Grooved, Biomimetic) and task (single or dual task) as intra-participants factors. Significant effects (statistical threshold of p ≤ 0.05) were further analysed using Newman-Keuls post-hoc tests.

We provided the partial eta squared values (η^2^) to estimate the effect size of the different effects that had the independent variables on the dependent variables (i.e., shear forces, postural reaction and cortical activity)^77^.

## Data Availability

The datasets generated and analyzed during the current study are available from the corresponding author on reasonable request.
